# Stool and Plasma Metabolites and Cognitive Function in the Boston Puerto Rican Health Study

**DOI:** 10.1002/alz70856_105357

**Published:** 2026-01-08

**Authors:** Deepika Dinesh, Thomas Kuntz, Xochitl Morgan, Tammy M Scott, Mahdi Garelnabi, Katherine L Tucker, Curtis Huttenhower, Natalia Palacios

**Affiliations:** ^1^ Department of Public Health, University of Massachusetts at Lowell, Lowell, MA, USA; ^2^ Harvard T.H. Chan School of Public Health, Boston, MA, USA; ^3^ Harvard T.H. Chan School of Public Health, Boston, MA, USA; ^4^ Tufts University, Boston, MA, USA; ^5^ Department of Biomedical and Nutritional Sciences, University of Massachusetts Lowell, Lowell, MA, USA; ^6^ Harvard T. H. Chan School of Public Health, Boston, MA, USA; ^7^ Department of Public Health, University of Massachusetts Lowell, Lowell, MA, USA; ^8^ Univ. of MA, Lowell, Lowell, MA, USA

## Abstract

**Background:**

Several studies have reported alterations in the gut microbiome and plasma metabolome in dementia, but no studies have concurrently examined metabolites in stool and plasma. Evidence is especially lacking in Latinos/Hispanics who have unique metabolic and microbiome profiles and are at an increased risk of dementia.

**Methods:**

Our study focused on 314 Boston Puerto Rican Health Study participants (median age 68 y), who provided concurrent stool and blood samples (untargeted metabolomic profiling, *Metabolon, Inc*) and underwent a comprehensive cognitive assessment, summarized as a global cognitive score (GCS). Multivariate analyses in MaAsLin2 were used to identify stool and plasma metabolites associated with GCS. Overrepresentation analysis (ORA) was used to identify metabolite sets associated with GCS. An FDRp cutoff of < 0.25 was considered significant.

**Results:**

We identified 14 stool and 257 plasma metabolites (89 at FDRp < 0.05) associated with GCS. In stool, palmitoleoyl ethanolamide (pFDR = 0.15) and steviol (pFDR = 0.20) were associated with better cognition. Lactobacillic acid (pFDR = 0.09), a microbially derived metabolite, stigmasterol (pFDR = 0.09), and other metabolites were associated with worse cognition. In plasma, gulonate (pFDR= 0.00007), N.acetylneuraminate (FDR= 0.0001) and many others were associated with lower GCS. Ergothioneine, an antioxidant, (FDR = 0.02), among other metabolites, was associated with higher GCS. ORA analyses identified that metabolites within the tyrosine metabolism group pFDR = 0.2145) in plasma, and long chain saturated fatty acid (pFDR = 0.0003) and tocopherol metabolism (pFDR = 0.0018) groups in stool, were associated with GCS more frequently than expected. Correlations between stool and plasma metabolites were limited and mostly restricted to caffeine‐related metabolites.

**Conclusion:**

Our findings of an association between the plasma metabolome and GCS are consistent with prior studies. More work is needed to examine the stool and plasma metabolomes in dementia.

